# Towards the Performance Characterization of a Robotic Multimodal Diagnostic Imaging System

**DOI:** 10.3390/jimaging11050147

**Published:** 2025-05-07

**Authors:** George Papaioannou, Christos Mitrogiannis, Mark Schweitzer, Nikolaos Michailidis, Maria Pappa, Pegah Khosravi, Apostolos Karantanas, Sean Starling, Christian Ruberg

**Affiliations:** 1 New Bedford Research & Robotics, Downtown New Bedford, New Bedford, MA 02740, USA; 2 Office of the Vice President of Health Affairs, School of Medicine, Wayne State University, 1241 Scott Hall, 540 E. Canfield, Detroit, MI 48201, USA; 3 School of Mechanical Engineering, Aristotle University of Thessaloniki, 54124 Thessaloniki, Greecepappmari@auth.gr (M.P.); 4 Biomedical AI, New York City College of Technology, Graduate Center, CUNY, 285 Jay Street, Brooklyn, NY 11201, USA; pkhosravi@citytech.cuny.edu; 5 Department of Medical Imaging, University Hospital, 71500 Heraklion, Greece; 6 Level 1, Meat and Livestock Australia, 40 Mount Street, P.O. Box 1961, North Sydney, NSW 2059, Australia

**Keywords:** robotics-driven imaging device, multimodal, radiation dose, dynamic accuracy, calibration, X-ray CT, tomosynthesis, stereovideoradiography, pediatric imaging

## Abstract

Characterizing imaging performance requires a multidisciplinary approach that evaluates various interconnected parameters, including dosage optimization and dynamic accuracy. Radiation dose and dynamic accuracy are challenged by patient motion that results in poor image quality. These challenges are more prevalent in the brain/cardiac pediatric patient imaging, as they relate to excess radiation dose that may be associated with various complications. Scanning vulnerable pediatric patients ought to eliminate anesthesia due to critical risks associated in some cases with intracranial hemorrhages, brain strokes, and congenital heart disease. Some pediatric imaging, however, requires prolonged scanning under anesthesia. It can often be a laborious, suboptimal process, with limited field of view and considerable dose. High dynamic accuracy is also necessary to diagnose tissue’s dynamic behavior beyond its static structural morphology. This study presents several performance characterization experiments from a new robotic multimodal imaging system using specially designed calibration methods at different system configurations. Additional musculoskeletal imaging and imaging from a pediatric brain stroke patient without anesthesia are presented for comparisons. The findings suggest that the system’s large dynamically controlled gantry enables scanning at full patient movement and with important improvements in scan times, accuracy, radiation dose, and the ability to image brain structures without anesthesia. This could position the system as a potential transformative tool in the pediatric interventional imaging landscape.

## 1. Introduction

Radiography (two dimensional—2D), computed tomography (CT, three dimensional—3D) fluoroscopy (2D and 3D) and tomosynthesis are different imaging modalities performed on separate dedicated diagnostic hardware. Although these modalities provide considerable morphological and temporal anatomical information for certain pathologies they require regular calibration for proper performance. All these different modalities are also yet to be combined and fused in one hybrid imaging system. Some imagers (O-Arm) [1] offer both 2D, 3D, and 4D modalities but the 2D and 3D data are not fused, and it is up to the operator to manually “combine” visually the four-dimensional (4D) effect by looking at the separated datasets in different monitors from different imaging sessions [2,3,4]. In addition, CT and Magnetic Resonance Imaging (MRI), are not yet capable of achieving the high frame rates required for estimating dynamic function (free of motion artifact or blurriness), i.e., anesthesia-free scanning options during voluntary movement [5]. Their fixed small “gantries” limit the overall field of view (FoV), making scanning of large non-axisymmetric parts of the human anatomy during increased range of movement very challenging. The effects of the geometry–angle of penetration of the X-ray signal and its exact diffractive/absorbing/scattering effect from individual hard and soft tissues are also constantly reassessed for the new generation of scanners. This conventional fixed scanner geometry also limits the capacity to automatically and dynamically alter the focal spot and control magnification with respect to amplitude, exposure, and the position of the patient relative to the positions of the emitter and the detector [6,7,8,9]. A conventional CT is designed to acquire images using fixed geometry with circular rotating rings and small gantries, with the patient required to be motionless. State-of-the-art micro- and nanotomography capabilities are also limited to very small, extensively prepared samples in situ, making these techniques not applicable for in vivo clinical diagnosis. Other image calibration studies reported additional imaging challenges including background luminance, viewing distance, line orientation, the frequency dependence of attenuation, scattering, spatial resolution (or sharpness), and contrast-to-noise ratio (CNR). Imaging is often confronted by issues such as tube-current modulation, radiographic mottle (quantum mottle effects), distortion, metal artifacts, and signal non-linearities that have been related to the tissue variations in size, the state of the recrystallization in bone or callus, and other tissue density characteristics [2,3,4,5,6,7,8,9,10,11,12,13,14,15,16,17,18,19,20,21,22,23,24,25,26,27,28,29,30,31,32,33,34,35,36,37,38,39].

In relation to dosage, there are several studies characterizing the relationship between the number of CT examinations and the lifetime attributable risk of cancer [2]. Significant overuse of radiation from radiological imaging processes has been reported for both adults and pediatric patients [4,10,11]. In pediatrics, in particular, radiologists must continually think about reducing exposure as low as reasonably achievable (ALARA), by using exposure settings customized for children, on a case-by-case basis, particularly for brain trauma patients. It has been reported that of the 600,000 abdominal and head CTs performed on patients less than 15 years of age, approximately 500 might ultimately die from cancer attributable to CT radiation [8,9,12,13,14,15,16,17,18,19,20,21,22,23,24,25,26,27,28,29,30,31,32,33]. The number of CT scans required to give a cumulative dose of 50–60 mGy depends on the type of CT scan, the age of the patient, and the scanner settings. If typical current scanner settings are used for head CT in children, then two to three head CT scans would result in a dose of 50–60 mGy (or mSv) to the brain. Similar red bone marrow dosage has been reported for children under 15 with five to ten head CT scans using current scanner settings [3]. Other studies [8,9,12,13,14,15,16,17,18,19,20,21,22,23,24,25,26,27,28,29,30,31,32,33] have drawn interesting corollaries between the widespread use of X-rays, reporting a statistically significant, increased risk of fatal cancer from low-dose radiation in the range of 50 to 100 mSv (mGy) which is the dosage in the majority of pediatric CTs in the state of the art. It was noted that a single CT of the abdomen could provide a dose of 11 mSv. It is therefore necessary that proper calibration, dosage control, and weight bands are employed for establishing safe pediatric diagnostic reference levels (DRLs) for trunk and brain radiography [11].

According to the European Society of Pediatric Radiology’s (ESPR) [19] strategic clinical agenda, academic pediatric facilities use lower CT radiation dose with less variation than nonacademic pediatric or adult facilities. The nonacademic centers being by far many more in number. The mean size-specific dose estimate (SSDE) for the smallest patients in nonacademic adult facilities is 51% (6.1 vs. 11.9 mGy) of the facility’s adult dose, which is almost double compared to academic hospitals [2,3,4,5,6,7,9,12,13,14,15,16,17,18,19,20,21,22,23,24,25,26,27,28,29,30,31,32,33]. Therefore, patient-specific controlled radiation dosage with finely tuned calibrated devices that can scan a patient without anesthesia has become an increasingly important issue for patients, surgeons, and the operating theatre staff.

It has been reported that there is a higher risk of tissue damage with anesthesia in pediatric populations with intracranial hemorrhages, brain strokes, and congenital heart disease in infants. Imaging intracranial hemorrhages and strokes requires the administration of anesthesia. Anesthesia, however, has been associated with a plethora of neurological degenerative effects in the developing brain in young children. Anesthesia is also associated with a high morbidity rate (16.5%) in pediatrics [27]. Other sources report stroke as the sixth leading cause of death in children; pediatric stroke is more prevalent than most people think, affecting 25 in 100,000 newborns and 12 in 100,000 children under 18 years of age [13,14,15]. The management of pediatric ischemic stroke is often challenging because of pleomorphic age-dependent risk factors and etiologies, the high frequency of subtle or atypical clinical presentation, suboptimal imaging, high radiological dose over short time, and a lack of evidence-based data about acute recanalization therapies [33]. This may exert long-lasting adverse effects on the individuals, their families, and society. The same challenge burdens the cancer or heavy-trauma pediatric population as they often require multiple revisions and surgeries with the disadvantage of the accumulative effects of additional radiographic imaging [33]. There is a substantial clinical need to improve the CT efficacy of these vulnerable groups by using the highest possible diagnostic standards [33,39]. The high risk associated with short- and long-term neurodegenerative effects in pediatric patients can be completely eliminated by removing anesthesia. This can be a huge asset when repetitive scanning in post-operative follow up is necessary. No imaging solution to date tackles all these challenges as it requires excessive patient-specific calibration for image quality, dosage, and resolution [16,17]. It has been suggested that hybrid robotic imaging devices can offer flexibility with a large field of view, controllable dosage options, and versatility in fusing 2D and 3D imaging in one view [6,9]. Detailed instructions for common tests are not provided here, as this information is widely available [7,17]. Instead, the objective here is to introduce new alternative calibration methods, so that the performance of a new hybrid robotic imager can be characterized. Special focus is given to the system’s unique features that can enhance pediatric imaging, especially in terms of lowering radiation exposure and eliminating anesthesia.

Therefore, the purpose of the study was to perform a series of novel calibration experiments designed to characterize radiation dose options in relation to improved image quality and the identification of soft- and hard-tissue boundaries. These boundaries ought to be identified during static imaging and imaging during patient movement. The work documents the ability to acquire CT data using multiple different X-ray sources during a single acquisition and without anesthesia. This, in turn, offers opportunities to demonstrate the use of the robotic scanner for new clinical applications. These applications include high-speed motion-compensation CT, dual-energy virtual non-contrast brain imaging, multiplane interventional radiography, and the assessment of tissue kinematics during movement. In parallel, automated dosage optimization and improved temporal resolution for brain and cardiac imaging are presented. We also discuss certain performance metrics of the system with respect to its complexity.

## 2. Materials and Methods

### 2.1. System Overview and Data Acquisition

**Figure 1:**
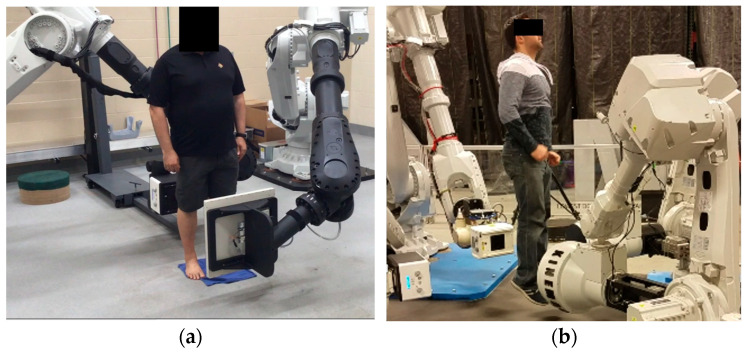
The “one-device–multiple-modalities” principle of the robotic scanner. (**a**) Patient’s 3D CT scan during standing and load bearing; (**b**) patient being scanned during load bearing and high-speed movement (jumping) with a set of multiple and different emitters/detectors.

There are several clinical and basic research studies associated with this robotic imager presented by our team elsewhere [36,37,38,39,40,41,42,43,44,45,46,47,48,49]. We refer to some of these studies by adopting previously published data for comparison with the calibration methods presented here (unpublished patient data with *n* = 10 adopted and re-analyzed from previously partially published work from our team). The Kinemagine Achilles System (KINEMAGINE/ATLAS Inc., New York, NY, USA [42]) employs several (6) six- to twelve-axis coordinated robotic (or cobotic) systems programmed with Programmable Logic Controller (PLC). The PLC tool communicates with the operator through a pedant. Through the pedant, the user sends instructions to the PLC to carry out scanning protocols with up to four combinations of X-ray housing units, dynamic flat panels, multicamera vision systems, intensifiers with high-speed radiography cameras, and densitometry detectors (Figure 1). In addition to the pedant, the system uses a Human–Machine Interface (HMI) device and End-of-Arm toolkits (EoAT) [46] to exchange different emitters and detectors. It therefore employs different imaging modalities and expresses their multimodal data in the same robotic global coordinate system. The system uses different robotic arm trajectories to meet the requirements of the size of the patient. Programmable adaptable collimators are placed after the emitters, in synergy with gridded X-ray generators and automated positioning of the focal spot. This integration enables the automated control of the exposure and FoV which, when combined with the right robot trajectories, can lead to an overall controlled and optimized emission for a patient-specific protocol.

**Figure 2:**
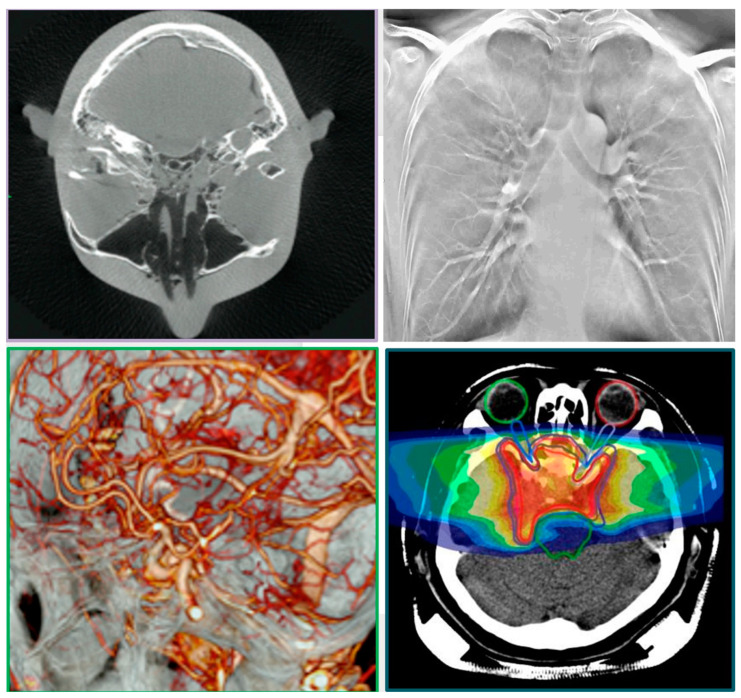
Different types of imaging modalities from the different scanning options of the robotic device. The “one-device–multiple-modalities” principle and data fusion results also from different robot trajectories leading to multiple modalities, i.e., digital radiography (DR-C), panoramic and 360 DR, 3D tomosynthesis, high-definition microCT, 3D stereovideography/multiplane CATHLAB).

**Figure 3:**
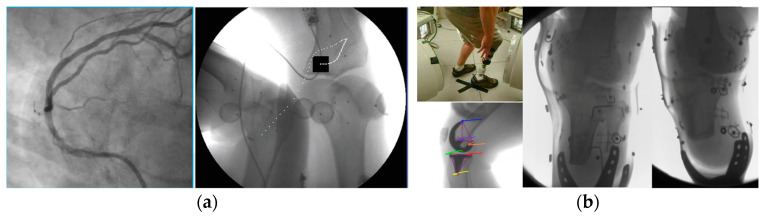
(**a**) High-speed stereovideoradiography cardiac angiogram (**left**) and dynamic assessment of arthrokinematics (**right**); (**b**) examples of scanning adopted from [41,45,48] presenting a jumping below-knee amputee patient (**top left**), tracking of an internal knee prosthesis (**bottom left**), and the two corresponding amputee videoradiography views recorded during the jumping task (**far right**).

Geometric and optical magnification techniques are also used to enhance resolution, a fact that can alter the slice thickness (ST). Therefore, the different ST or the overall number of frames collected (as, for example, in the case of the CT number of water vs. ST) is always reported. The “one system–many (potential) modalities” options offers the error-free fusion of all the imaging modalities and co-registration of all the data using commercial software (3Dnet v1-Biotronics 3D Boston and ANSA-ANSA BETA CAE systems Switzerland) [11,38,39,42,43,46,50,51]. The modalities (Figure 2 and Figure 3) available in this study were: 2D single-plane radiography, panoramic radiography, computed tomography (CT), dynamic imaging similar to CATHLAB but with multiple views (3D stereovideoradiography with more than two detectors), tomosynthesis (high resolution magnified 3D imaging), and microCT [37,38,39,41,42,43,44,45,46,47,48,49,52].

**Figure 4:**
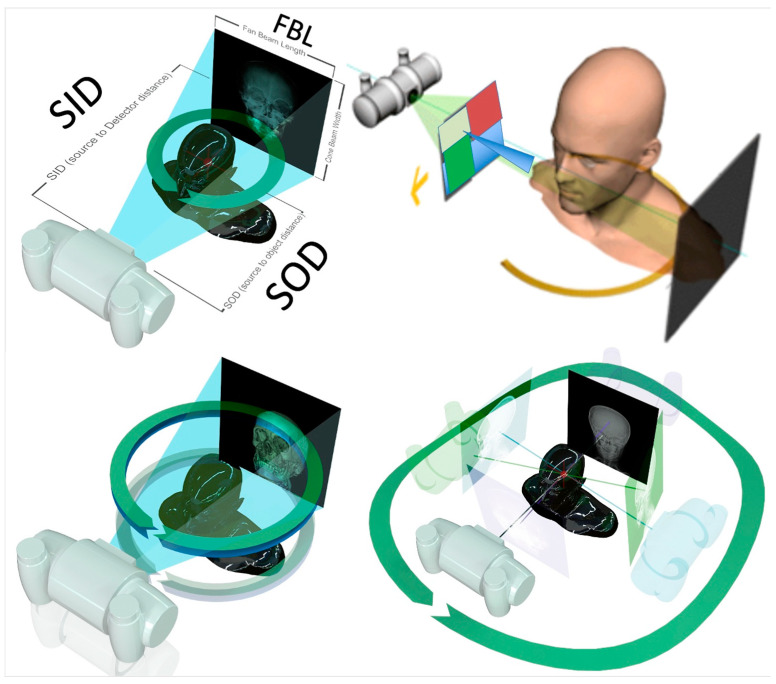
(**Top left**) The capacity of the robots to move in different pathways 360° around the subject and the definition of the source-to-object distance (SOD) and source-to-imaging detector distance (SID); this functionality produces a patient-specific collimation (**top left**) and FoV depending on the target.

In Figure 4, the bottom left drawing shows the ability of the robots to complete helical swipes around the subject for the entire length of the human body at homocentric cycles; the bottom right drawing shows the unique capability of the robotic scanner to change the trajectories of the robotic arms away from the homocentric paradigm; Figure 4’s top right drawing shows programmable adaptable collimators using four programable shielding plates working in synergy with the position of the panels for different types of volumetric scanning (from short scans to “fan” type of scans). Synergy is also programmed with the gridded X-ray generators and the adaptable collimators to control the exposure and respond to changes in focal spot (FS). This functionality produces a specific FoV leading to an overall controlled and optimized emission for a patient-specific protocol.

**Figure 5:**
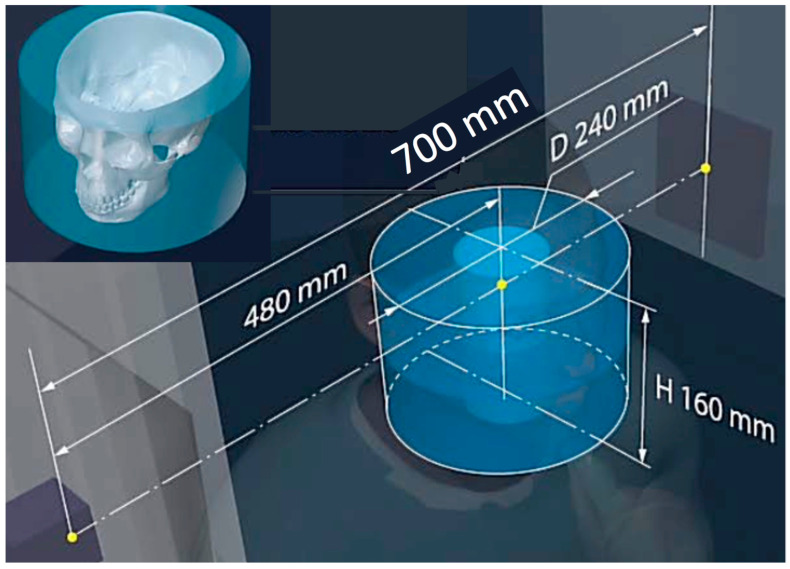
The unique capability of the robotic scanner to change the trajectories of the robotic arms away from the homocentric paradigm is assisted by the programmable adaptable collimators and the ability to position the panels in the right geometric relationship with the patient for different types of volumetric scanning (from short scans to “fan” type of scans).

**Figure 6:**
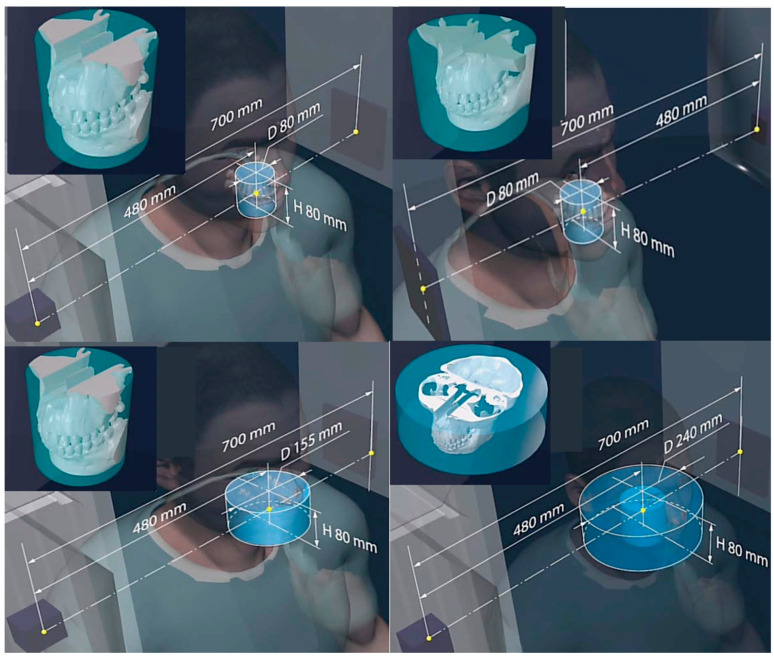
The capacity of the robots to move 360° around the subject, and the unique capability of the robotic scanner to change the trajectories of the robotic arms away from the homocentric paradigm enables different types of synergies for various patient-specific protocols for appropriately chosen volumetric reconstruction in 3D.

The principle of the device’s specialty collimation technology and the resulting geometrical parameters for optimized volume reconstruction are presented in Figure 4, Figure 5 and Figure 6. Synergy is also programmed with the gridded X-ray generators to control the exposure, focal spot (FS), and FoV leading to an overall controlled and optimized emission, for a patient-specific protocol such as the one shown in Figure 5. Figure 5 depicts the scanning option called 3x mode combined with the Stacked Volume mode with a 700 mm source-to-imaging detector distance (SID) and 480 mm source-to-object distance (SOD) that produces a volume with a 240 mm conical volume diameter (VD) and a 160 mm conical volume height (VH), at a scan angle of 1440° and focal spot (FS) of 0.06 mm. The individual reconstructed 3D CT of the various parts of the head is also shown in Figure 5, demonstrating the effect of some of the geometry parameters on increasing the reconstructible CT volume. The robotic arm can produce multiple and different trajectories and, in combination with appropriately chosen collimation, alter these parameters for optimized volume reconstruction. The additional enclosed drawing in Figure 5 displays the resulting size of the head anatomy that will be included in the respective reconstructed volume. Some more examples for head imaging volume reconstruction are presented in Figure 6. The capacity of the robots to move 360° around the subject, and the unique capability of the robotic arms to move the detector–emitter combination away from the homocentric pathway enables different types of synergy for various patient-specific protocols. Clockwise from top left in Figure 6, the first drawing shows the option for a full CT scan with a 700 mm source-to-imaging detector distance (SID) and a 480 mm source-to-object distance (SOD) that produces a cylindrical reconstructible volume with an 80 mm diameter (VD) and 80 mm height (VH), a scan angle of 360°, and a focal spot (FS) of 0.3 mm). The next option is for a conventional short CT scan with a 700 mm SID and 80 mm SOD that produces a volume with an 80 mm VD and 80 mm VH, a scan angle of 200°, and an FS of 0.6 mm. Next is the option for a 3x mode CT scan (720°) with a 700 mm SID and a 480 mm SOD that produces a volume with a 240 mm VD and 80 mm VH, a scan angle of 720°, and an FS of 0.02 mm. The final option is a half-beam mode CT scan with a 700 mm SID and a 480 mm SOD that produces a volume with a 155 mm VD and 80 mm VH, a scan angle of 360°, and an FS of 0.6 mm. All drawings enclose the display of the resulting size of the head anatomy that will be included in the respective reconstructed volume.

### 2.2. Emitters and Detectors

The different X-ray generators that were used in the present work had the following specifications [52]: max voltage of 1–160 kV with max power of 300 W–1.2 kW and max current of 300 mA. Note that a wide variety of focal spots were implemented (ranging for 0.063 to 7.5 mm and microlevel applications with the microfocus focal spot sizes reaching 16 μm; the automated focal spot alterations are a unique functionality in this system). Detectors included amorphous silicon (a-Si), indium gallium zinc oxide (IGZO), CMOS, and perovskites-based sensor-type panels. The specifications of the three detectors included the following options: (a) an amorphous silicon flat-panel detector (FPD) of 43 × 43 cm, pixel matrix of 3072 × 3072, pixel pitch of 139 μm, pixel size of 45 μm resolution, limiting resolution at low-power mode of 3.6 lp/mm, energy range of 40–150 kVp, radiation tolerance of 2000 Gy, and max frame rate of 40 fps; (b) an IGZO (indium gallium zinc oxide) sensor detector of 16 × 16 cm, pixel matrix of 1536 × 1536, pixel pitch of 135 μm resolution, and max frame rate of 160 fps; (c) a perovskite 30 × 30 cm detector with sensitivity of 1.1 × 104 μC·Gyair^−1^·cm^−2^, spatial resolution of 11 line pairs per millimeter (lp mm^−1^), demonstrating high X-ray sensitivity of 20,570 μC Gy^−1^cm^−2^, a lowest detectable dose rate (LDDR) of 0.98 nGy s^−1^ and a fast response time (154/162 ns), the ability to endure strong dose rates (high dose rate of about 100 μGy s^−1^ and low dose rate of about 20 nGy s^−1^) for over 300 min if needed. Binning and automatic stitching of images was offered and could positively affect the resolution and FoV.

Coupled with the image intensifiers’ configuration were specially customized (CMOS) Back–Side-Illuminated (BSI) sensors (cameras with extreme dynamic range-EDR) with a 9.27 µm pixel size, (2560 × 1664)-pixel resolution, and capacity for 9350 fps. These two cameras could stereotactically scan the patient from two different robotic arms during the patient’s movement. Using these stereoscopically high-speed cameras helped resolve challenges with image artifacts (blurriness), contrast, noise, and motion detection compensation in this system as demonstrated in previous studies [37,38,39,42,43,44,45,46,47,48,49]. External fiducial markers (0.1 mm tantalum beads) attached on the surface of the patient or phantom to be scanned are used as recommended in many of these stereovideoradiography approaches involving the device [38,39,45,47]. The system processes 450 to 12,000 2D projections in real time, depending on the application and mode, providing 3D reconstructions in almost real time. Using the pulsing X-ray capabilities, the accumulative exposure time can be much less than a millisecond (<1 ms) and up to several minutes in prolonged scans. The highest accuracy is obtained with the highest repeatability option of the robots tuned to 0.005 mm for translation of the robotic arms at less than 1 degree per second for the respective robotic arm angular velocity.

### 2.3. Calibration

Several calibration tests for dosage and image quality assessment were performed. Specialized software was used for the analysis of the data such as 3DNet medical cloud with AI-driven workflow PACS software (3DNet v1), ImageJ (IJ 1.46r), and ANSA BETA CAE systems (2018 version). The specific hardware utilized in the calibration studies included the KINEMAGINE distortion correction, radio-calibration frame, and robot geometry alignment cube, the ACR 464, Catphan 500 CT and PBU-70 phantoms, a DAP meter, several resolution test pattern tools and a 5 cc ion chamber. The following calibration tests were performed:

(a) Distortion correction, while field imaging baseline and system geometric alignment with local and global coordinate systems;
(b) Calibration for cupping effects, scatter correction (SC), streaking, and beam-hardening correction (BHC);
(c) Radiation dose measurements with a specialized custom frame that included a pediatric whole-body phantom;
(d) Dose–area product (DAP) measurements for the fluoroscopy mode;
(e) Comparative burst progressive pulse fluoro (BPPF) mode radiographic dose experiment;
(f) Radiologic dose experiments and their relationship to X-ray power and energy generation;
(g) Over-and-under-exposure imaging for diagnostic quality.

**Figure 7:**
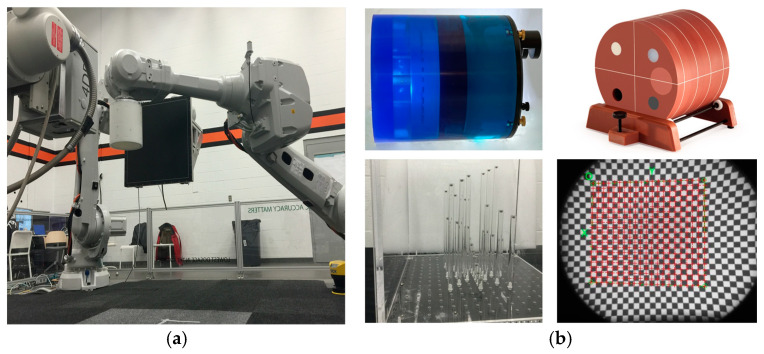
(**a**) The robotic calibration setup utilizes three robots. The two robots carry the detector and emitter each time, while the third one is used to accurately position the different phantoms within the field of view; (**b**) clockwise from top left: the ACR 464 CT and Catphan 500 CT phantoms; custom-made 1145-marker 3D cube phantom/grid for distortion correction and geometry alignment (**bottom left**) and a sample grid distortion correction image (**bottom right**).

**Figure 8:**
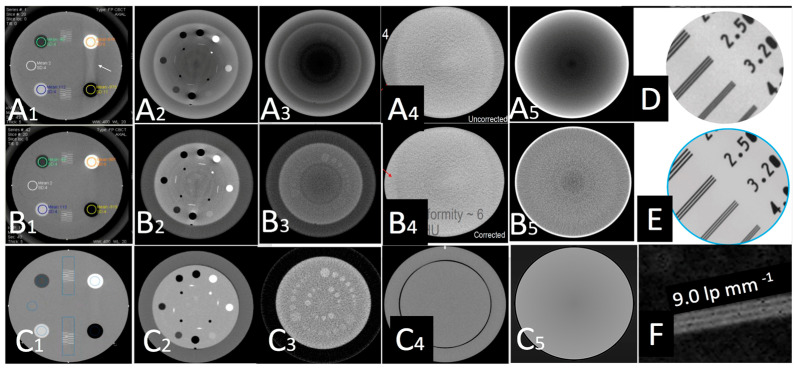
The calibration experiments using the ACR 20 cm CT phantom included scatter correction that removed the artifacts between air and bone plugs (**A1**,**B1**), the cupping artifact (**A2**,**B2**), and also improved the low-contrast plugs (**A3**,**B3**); detector options; (Uncorrected and corrected uniformity scans (**A4**–**C4**) and the cupping artifact correction of SC and BHC (**A5**–**C5**) for the three detector options, respectively. The resolution test pattern differences of the three detectors options are shown in (**D**–**F**), respectively.(**C1**–**C3**) always presented the most improved results.

Each calibration experiment is presented in detail next:

(a) We initially performed distortion correction, while field imaging baseline tests and system geometric alignment with local and global coordinate systems. Two custom-made 1145 tantalum marker 3D grids (Figure 7) were used to correct (a) the distortion introduced by the image intensifiers and associated optics and (b) align the geometry of the tissue kinematics tracking software with that of the robotic hardware as prescribed by the manufacturer [52]. Additionally, a white-field image (acquired from the X-ray system with nothing in the FOV) was used to perform a log-based correction for image intensity nonuniformity.
(b) The second type of calibration used an ACR 464 CT (Sun Nuclear) and a Catphan 500 CT (Phantomlab) phantom (Figure 7). This calibration included the correction of the issue of scatter-to-primary ratios whereas optimal large cone angles and large detector apertures were chosen in those tests. These tests reported (a) cupping in the Hounsfield Unit (HU) profiles of uniform objects and (b) streaking between high- and low-density objects. For mitigation and correction, the device used software-based stepwise mechanical corrections, an anti-scatter grid on the detector, a bowtie filter at the source, and a protocol-based extension of the patient-to-detector distance based on previous testing with a specialized custom frame (Figure 7b) [52]. The calibration included projection normalization with water scans, empirical scatter kernel corrections on projection data, and scatter correction using a deconvolution method (results were reported on uniformity of ±7 HU across a 20 cm water equivalent phantom) (Figure 8). A third resolution evaluation with resolution test pattern tools (2 to 20 lp/mm) was conducted for all three optional detectors (Figure 8D–F).

(c) Another set of calibration and radiology dose tests included a pediatric whole-body phantom (PBU-70, Kyoto Kagagu Ltd., Kyoto, Japan), which consists of tissue-equivalent materials for bone and soft tissues in a 5-year-old. This was located on a specially designed frame [52] to emulate the irradiation received and scatter caused by the patient’s body with respect to the X-ray source. This custom calibration frame allowed us to test for an unlimited choice of distances from the source to the phantom to the detector including the cross point of the mid-transverse, mid-coronal, and mid-sagittal planes. Several different source-to-detector configurations and distances were investigated.
(d) To assess the output of the X-ray source in the fluoroscopy mode, the dose–area product (DAP) was also measured. A DAP meter (VacuTec, Dresden, Germany) and/or Accu-Dose+ (Radcal, Monrovia, CA, USA) was placed on the inner surface of the robotic trajectories and attached to the X-ray tube. A total of 5 min of radiation was applied and averaged into the final DAP measurements. The air kerma in the middle of the X-ray beam was also measured by placing a 5 cc ion chamber (Capintec, PM-05, Cambridge, MA, USA) at the center of the field of view along with the measurement of scatter radiation in the surrounding area of the patient at equally spaced polar coordinate grids. The air kerma rate was measured as a scatter radiation dose using an 1800 cc ionization chamber during 5 min of continuous use imaging. This measurement was performed at horizontal distances of 40, 60, 80, and 100 cm from the xyphoid process at angle positions of 0, 45, 90, 135, 180, 225, and 270°. The tube position was anterior posterior (AP), and the distances between the patient phantom and the tube in the robotic system were 65 cm.
(e) A very simplified, one-source-only-each-time dosage experiment was also designed to demonstrate the fundamental characteristics of the burst progressive pulse fluoro (BPPF) mode. This mode was applied as a stereovideography mode with a minimum of two sources firing synchronously. The grid-control system was by default paired with the system’s grid-controlled X-ray tubes. When the system CPU commanded an X-ray exposure, a generator turned on the high voltage (kVp), but the tube current (and X-ray creation) was restricted by the presence of the previously applied negative high voltage (applied by the system CPU) upon the X-ray tube grid. Without this tube current, X-rays were not generated. This tube’s current modulation technology was utilized in this system to minimize radiation dose for patient safety. While the intention was to minimize patient dose, the technical objective was to create X-rays, when the imaging camera/detector was “looking”, and stop producing X-ray, when the camera was in “blinking-off status”. The physics of the energy involved dictated that high-speed X-ray/camera looking–blinking synchronization could only work with grid-control technology. This grid-control tube-current modulation technology was simply a way to control tube current. Tube current was commanded by the system CPU when it called for a specific length and quantity of X-ray signal releases (these releases came from the switching of the grid potential, from its previously applied negative voltage to zero volts, relative to the cathode). Each release lasted, at full X-ray potential, no more than 2 μs after the CPU’s command for said pulse was initiated. This super-fast “up” time was one of the ways the grid-control system produced the remarkable dose savings. Thereafter, the time for actual X-rays to reach the receptor was even shorter. After the initiation of these X-ray pulses, each pulse began to stop when commanded to do so by the system’s CPU. Note that this happened when the system commanded the reapplication of a negative voltage to the grid. This same sequence of events happened for each and every pulse (each frame).

**Figure 9:**
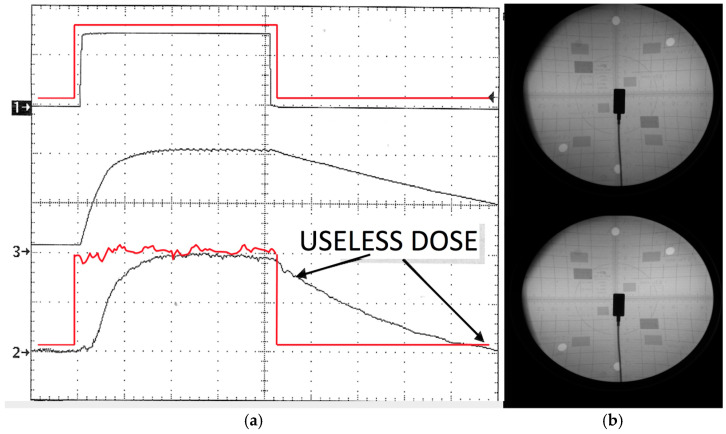
(**a**) In BPPF mode, the rise and fall times of each pulse were removed and the X-ray was also turned on and off multiple times within each pulse, where the red line indicates that the useless dose was removed from the system; (**b**) the top shows case (ii)’s gridded pulse, whereas the bottom image is BPPF with superior contrast and range; the densitometer is shown at the center of the detector.

For reference, see an example in Figure 9a, showing that upon the termination of each pulse, most radiation stops when the grid reaches −1 kVp and the time of this complete shut off of radiation is approximately 2 μs. The full −3.5 kVp is reached at the grid in approximately 15 μs (these values can vary). All the while, the generator’s kVp remains constant during all the multiple pulses. This super-fast “down” time is the primary way grid-control technology produces remarkable dose savings and removes nearly all “soft”, non-useful radiation (indicated as “useless dose” in Figure 9a). The sequence of turning on, then off, of the X-ray emissions by the grid-control robotic system can happen very fast—much faster than 30–120 frames per second in currently commercially available systems. The system operates at 5 kHz frequency and thus can be paired with a camera operating at high frequency (up to 5000 fps). Each and every one of the time gaps between frames is an opportunity to remove unwanted and unneeded X-ray dose. The amount of the dose that can be saved is directly linked to the amount of time that the X-ray is turned off between camera frames. Such savings can be very dramatic.

Some samples of irradiation and dose output were tested. The first configuration of the variant generator settings used for these tests had the following specifications: input voltage of 230 VAC single phase 40 kW, min current of 10 mA, max current of 500 mA, with a kVp range of 20–125 in 1 kVp increments, and a high-voltage ripple of less than 1% of the max kVp at all ranges. The on/off duty cycle was controlled by a command of 0–100% through the PLC system operating at a max frequency of up to 5 kHz. The radiation output’s rise time was 2 μs with a radiation fall time of approximately 2 and up to 15 μs (pulse widths as low as 1 μs with repeatability of 0.5%). If the system commanded 50 μs pulses and a frequency of 5 kHz, the grid automatically followed (slave) almost any such command (this is one example, and obviously other options can be chosen). The trial and error of different techniques and an interplay of such parameters was tested to determine how much exact dose’s “off time” would be present (and thus how much dose would be saved).

In summary, the dosage tests that were recorded for reference to compare the pure pulsed and cine fluoro modes along with the system’s burst progressive pulse fluoro (BPPF) were as follows: (i) a baseline standard pulse fluoro (no grid)– for a generator setting of 100 kVp/40 mA/30 fps and 4 kW was required to generate X-rays; (ii) for the same generator setting with the gridded-only mode, 4 kW was required to generate X-rays; (iii) for the same generator setting in BPPF mode, 2 kW was required to generate X-rays; (iv) a baseline standard pulse fluoro (no grid) for a generator setting of 100 kVp/25 mA/30 fps, and 2.5 kW was required to generate X-rays; (v) for the same generator setting, with the gridded-only mode, 2.5 kW was required to generate X-rays; (vi) for the same generator setting and the BPPF mode, 1.25 kW was required to generate X-ray.

(f) Additional dosage experiments were expanded to include the following: (Dose A): a 10 μs integration example with 40 mA/60 kV for 15 s in pulsed output fluoro mode using a 100% pulsed max dose, that is, a “non-gridded baseline” example; (Dose B): the same technique at 40 mA/60 kV/15 s run in pulsed fluoro mode at 30 fps, with 10 ms pulses (based on an assumed 10 ms integration grab time); (Dose C): the same technique, 40 mA/60 kV/15 s continuous output fluoro coupled with a high-data-acquisition-speed camera at 1 kHz and a 100% max dose; (Dose D): the same technique, 40 mA/60 kV with 15 s runs of pulsed fluoro at 1 kHz-Fps, 500 μs pulses (based on a 400 μs camera integration grab time). The same techniques were repeated for 30, 60, and 120 fps. Dosimetry was obtained in all these experiments.
(g) In this calibration tests, the pediatric phantom was used in over-and-under-exposure settings and the images were evaluated by a certified radiologist to systematize the correspondence between the tests’ gray-level histogram (calculated using ImageJ -ImageJ NIH, Version 1.54p 2024) and the diagnostic quality of the image.

We also performed an angiography scan after obtaining informed consent using the ALARA principle from one of the pediatric patients (enrolled in the study approved by the University of Wisconsin Milwaukee and Stony Brook Hospital IRB committees-[37,47]). The 5-year-old patient (female, height: 115 cm, weight: 26 kg) was brought in the emergency room with early stages of ataxia and acute hemiparesis after an intracranial arterial ischemic stroke incident. The scan was performed during the “golden” hour time limit for diagnosis and intervention. After that limit the risk for irreversible damage is higher. It has been reported that the therapeutic yield is maximal in the first minutes after symptom onset and declines rapidly during the next 4.5 h [13,14,15]). A follow up MRI–MRA (Circle of Willis) one-year post-stroke was also performed. All the images/datasets from the calibration studies, the dosage studies, and the in vivo pediatric respective data were analyzed using the 3DNet (v1) medical cloud and AI-driven workflow software (Biotronics 3D Inc., Boston, MA, USA) [52] and ANSA (version 2018, ANSA BETA CAE systems, Lucerne, Switzerland) [50] software modules for image quality, meshing, and Euclidian distance calculation between pixels/voxels. The data were compared with previously published diagnostic reference levels and achievable doses of pediatric radiography studies from the American College of Radiology Dose Index Registry.

It should be stressed that some of the methods adopted here were designed for the first time for a very unique hybrid robotic device with very different system architecture than that of other conventional scanners. In that sense, the scope of these methods was to indicate the trends and set the foundation for a new series of calibration procedures that are more appropriate for such a device. Having limited datasets that cover many domains of calibration did not allow us to demonstrate significance, a fact that needs to be addressed in our follow-up work. We believe, however, that the methodologies presented here are valid for all future investigators of similar robotic hybrid imagers. To the authors’ knowledge, there are no similar methodologies and datasets yet available in the literature.

## 3. Results

**Figure 10:**
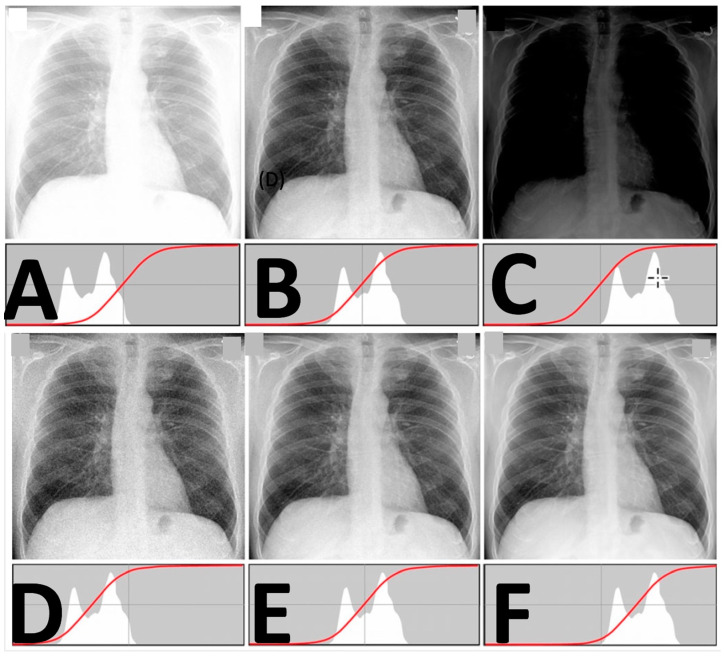
Dosage and diagnostic value experiments with the pediatric phantom were used in over-(**A**,**B**) and under-exposure (**C**) settings for evaluation by a certified radiologist; this systematized the correspondence between the tests’ gray-level histogram (brightness–contrast) and the diagnostic quality of the image. The values of (**D**–**F**) are from the BPPF so both image quality and dosage parameters could be optimized and used as baseline in the in vivo experiments.

**Figure 11:**
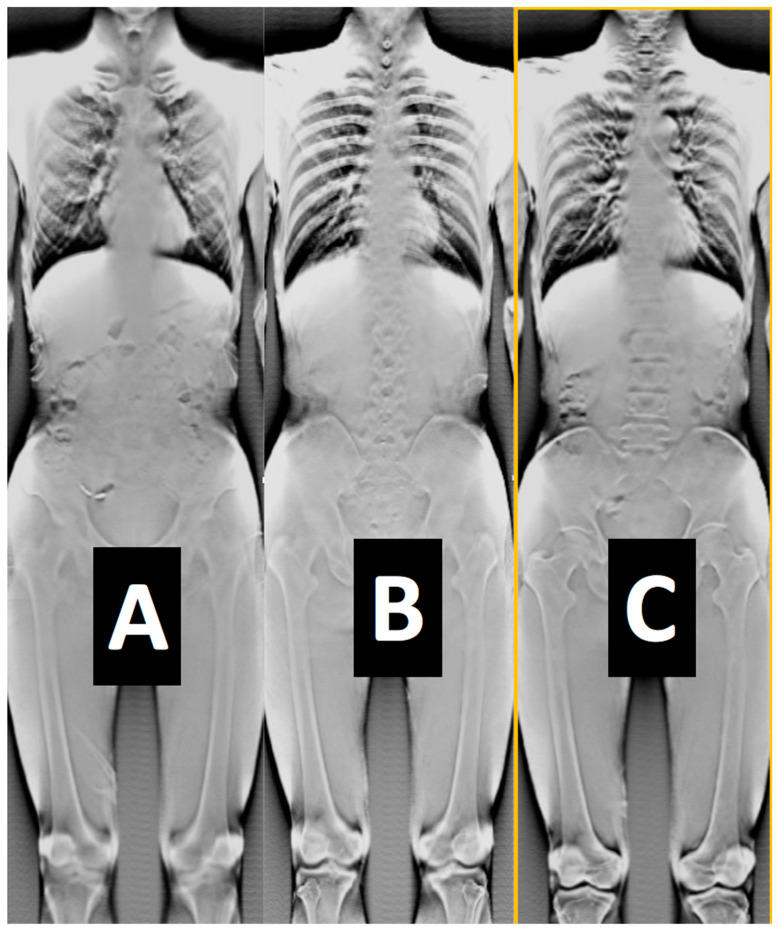
Tomosynthesis (40 μm) images using a dynamically adjusted focal spot (ranging from 0.1 to 0.02 mm) and the geometry for the reconstruction described in Figure 2 shown here as sequences of the whole body of a human standing for an otherwise narrow field of view (*z*-axis). Images (**A**–**C**) show three sequences of a full-body tomosynthesis scan with similar settings as the CT (Figure 6 top left) but with different reconstruction geometry, i.e., with a much larger SID of 1100 mm.

**Figure 12:**
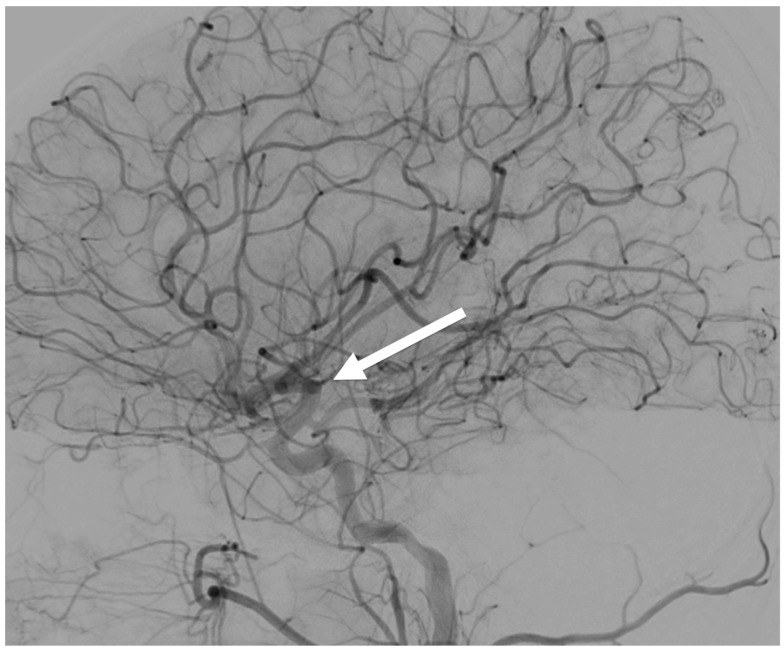
The in vivo angiography image from a 5 yr old pediatric patient that was obtained with the robotic scanner in CATHLAB mode. The data acquisition in the CATHLAB mode reached acquisition rates up to 10,000 Fps as the detectors rotated around the head. The CT 3D morphology of the tissue of the total organ and of the system of arteries and veins was reconstructed using the 3DNet vascular filter [51]. Once the 3D space was assigned, the dynamic tissue deformation and the blood flow data could be visualized synchronously.

**Figure 13:**
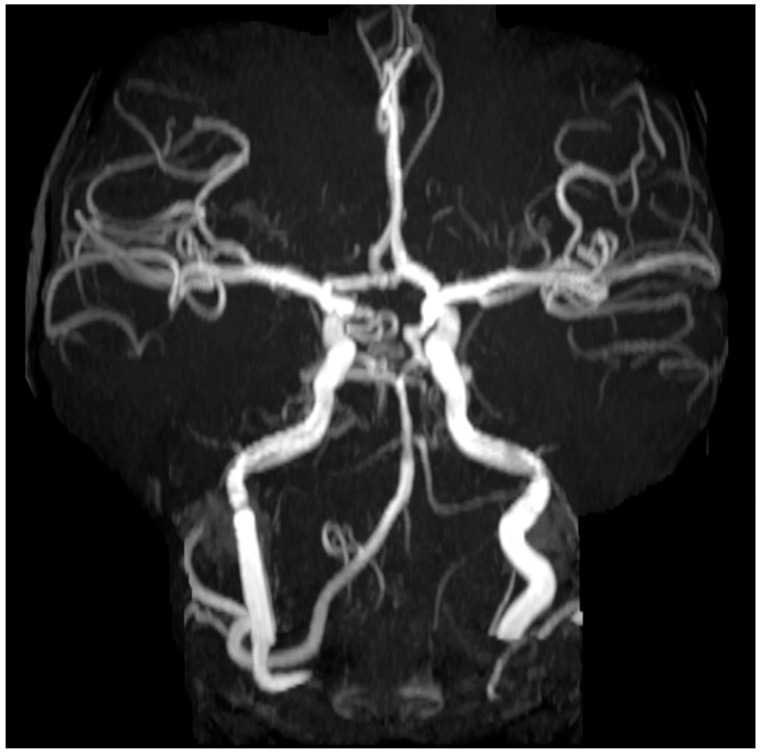
Magnetic resonance angiography (MRA) for the pediatric patient’s brain without IV contrast (SPIN: Circle of Willis (COW) spin) collected one year post-stroke.

Some examples of images from the multimodal imaging modalities that resulted from the relationship among the multitude of different robotic trajectories are presented in Figure 2, Figure 3, Figure 10, Figure 11 and Figure 12. These figures relate to imaging using high-speed motion compensation and the device’s specialty collimation/exposure for optimized volume reconstruction as explained in Figure 4, Figure 5 and Figure 6. The top left image in Figure 2 shows a patient head 3D CT being scanned during standing and load bearing (see Figure 5 for details on its reconstruction geometry). The top right image shows a patient’s lungs being scanned during breathing using 3D tomosynthesis with a different set of emitters/detectors (see Figure 6 top left, for the reconstruction geometry but with a fixed detector position). Three-dimensional tomosynthesis is a modality similar to but distinct from CT which uses a more limited angle in image acquisition (see also Figure 11A–C for full-body tomosynthesis with similar settings as the CT (Figure 6 top left) but with different reconstruction geometry, i.e., with a much larger SID of 1100 mm). This out-of-plane 3D tomosynthesis scan was only possible with the particularly long, wide, and appropriately elliptical trajectories of the emitters and detectors offered by the robotic arms. Rather than a 360-degree acquisition of a structure, tomosynthesis, via an X-ray tube “arcing” method over a stationary detector, is capable of capturing an arc sweep of a single part of the target. One of the primary advantages of tomosynthesis is its very high-resolution capabilities (as it functions as a magnification method in conjunction with dynamically altered focal spot). The bottom left image shows a high-definition brain 3D microCT using a 0.02 mm focal spot rendered to demonstrate the various arteries and capillary structure from the patient’s in vivo data also presented in Figure 12 and Figure 13. All the aforementioned images were collected without anesthesia and were adapted from our previously published datasets [44,48] and re-analyzed with new visualization and reconstruction methods. The new 3D reconstruction and visualization “sub-windows” plot (Figure 2 bottom right) method employed here used commercial software (3Dnet and ANSA [51,52]) with all data from all the modalities fused in one picture archive and communication system (PACS) window. These modules enable segmentation and meshing and highly accurate calculation of Euclidian distances between pixels and surfaces (reconstructed tetrahedra or other types of meshes from the volumetric data) in the 3D images that can be used for accurate anatomical and pathology measurements.

**Figure 14:**
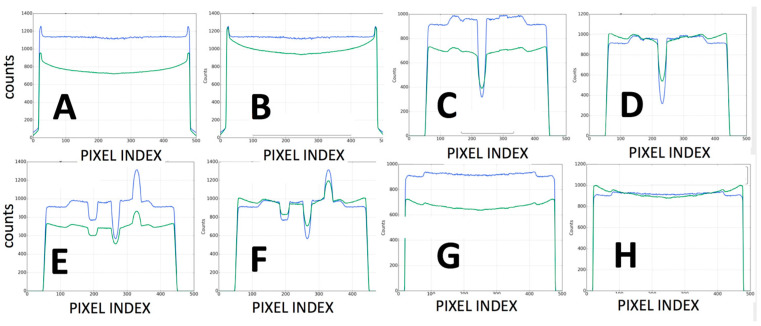
Graphs (**A**–**H**) are the plots from the calibration processes describing the number of counts versus pixel index for all the calibration experiments described in the methods. The graphs describe the effects that scatter (SC) correction and beam-hardening correction (BHC) had on the images of the ACR 464 CT and Catphan 500 CT phantoms used in the study.

**Figure 15:**
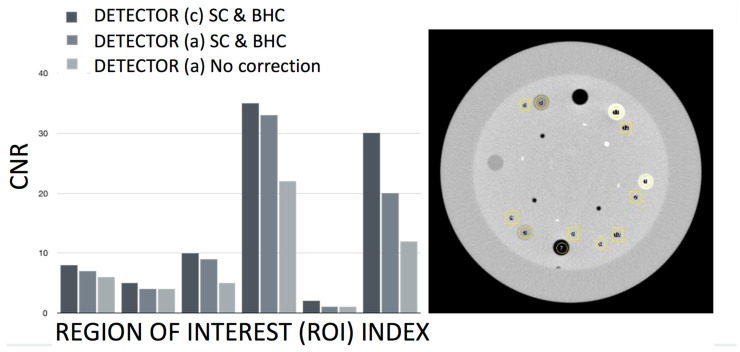
The improved CNR after SC and BH corrections shown left for detector option (a); On the **left**, the graph indicates the corrected vs. uncorrected CNR for detector option (a) and the corrected values for detector option (c), which presented the lowest CNR values (also shown in the image on the **right**).

Those datasets were obtained as a result of a series of calibration studies introduced in the present study and are, to the knowledge of the authors, the first level of characterization of the performance of this hybrid robotic imaging system to date; special attention was given to image quality for different modalities, dosage options, detector type, and X-ray power generation levels. The calibration experiments for the scatter correction (Figure 8) showed that artifacts between air and bone plugs (Figure 8(A1), using a 10:1 anti-scatter grid and polynorm provided by the manufacturer) on an ACR 20 cm CT phantom were completely removed with the system’s polynorm and scatter correction (SC) functions (Figure 8(B1)). The SC and beam-hardening correction (BHC) had a clear enhancement effect by removing the cupping artifact and improving contrast on the Catphan phantom (Figure 8(A2,B2) and Figure 14C–F, respectively). Figure 8(B2) was produced with the use of a bowtie filter. The effects of SC and BHC with the respective phantoms for detector option c) are shown in Figure 8(C1–C3), always presenting improved results. SC and BHC also improved the low-contrast plugs (Figure 8(A3,B3) and Figure 14G,H, respectively). Figure 15 shows the improved CNR after the corrections and Figure 8(A4,B4) show the uncorrected and corrected uniformity scans. Figure 8(A5,B5) show an additional cupping artifact correction of SC and BHC with the water phantom, respectively. It should be noted that the perovskite detector in the detector option (c) demonstrated the highest spatial resolution of ~9 lp mm^−1^ (Figure 8F) and excellent X-ray imaging properties. The detector presented this resolution under a remarkably low X-ray dose of ~56 μGyair, which was just half of the X-ray dose used in the other two detectors utilized in the tests. The spatial resolution of detectors option (a) and (b) were 3.2 and 3.9 lp mm^−1^, respectively (Figure 8D,E and Table 1). The system’s highest dynamic translational and rotational accuracy values were 10 μm and 0.02°, respectively (Table 1). An image of the fluoroscopy distortion correction process is shown in Figure 7b. All the additional phantom tests (Figure 8(C1–C5)) were also performed for the detector option (c) with much improved calibration imaging results.

Figure 14 shows plots from the calibration processes describing the number of counts versus the pixel index for all the calibration experiments testing the effects that SC and BHC had on the images of the ACR 464 CT and Catphan 500 CT phantoms. Figure 14A,B are the line profiles (100-slice average) showing that at ~200 HU the cupping artifact was removed by SC and BHC with the water phantom. Figure 14C,D) are line profiles (100-slice average centered at row 370) showing the reduced cupping artifact by SC and BHC with the Catphan phantom. Figure 14E,F are line profiles (100-slice average centered at row 128) showing the reduced cupping artifact by SC and BHC with the Catphan phantom. Figure 14G,H are line profiles (100-slice average centered at row 250) showing the reduced cupping artifact by SC and BHC with the Catphan phantom; SC and BHC had a very noticeable effect by removing the cupping artifact and improving contrast on the Catphan phantom (see phantom in Figure 7b) but also improved the low-contrast plugs (Figure 8B1–B3 and Figure 8C1–C3, respectively).

In another set of tests, the dose–area product (DAP) and air kerma at the center of the X-ray beam for the robotic system (detector option (a)) with the pediatric whole-body phantom (always at the anteroposterior (AP) position) was measured. They were found to be 1.4 mGy m^2^ min^21^ and 22.2 mGy min^21^, respectively. The respective values for the other two detector options (b and c) were 1.7 mGy m^2^ min^21^ and 29.3 mGy min^2^ and 0.7 mGy m^2^ min^21^ and 14.2 mGy min^21^. Other similar studies have reported DAPs for the O-arm being 1.53 mGy m^2^ min^21^ and 43.3 mGy min^21^, which are 2–3 times higher than that of the robotic system [4]. Similarly, the air kerma at the beam center of the robotics arm was 2.5 times lower than the air kerma in the O-arm. It should also be noted that the robotic system can cover angles, distances, and beam penetration orientations that the O-arm cannot deliver due to its fixed geometry. These differences are also attributed to the fact that the FOV of the O-arm is wider than that of the robotic system. In addition, the ratio of air kermas at the beam center was similar to the ratio of mA used in the two systems.

**Figure 16:**
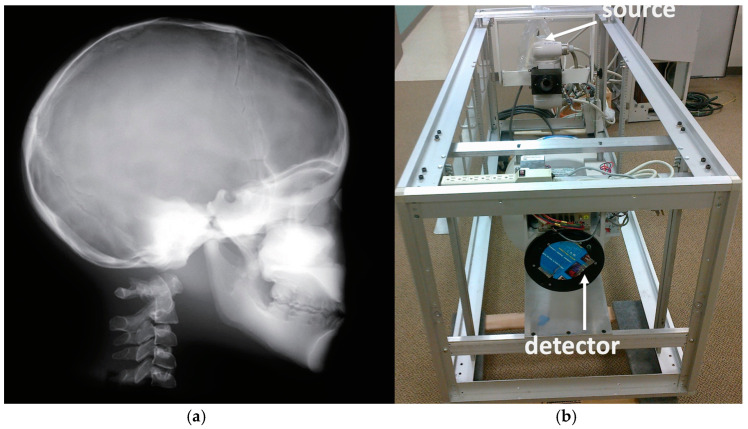
(**a**) Image using the BPPF scanning mode of the head of the PBU-70 pediatric phantom indicating a resolution result with high diagnostic value; (**b**) customized radio-calibration frame that allows accurate positioning of the phantom in the imaging equipment.

**Figure 17:**
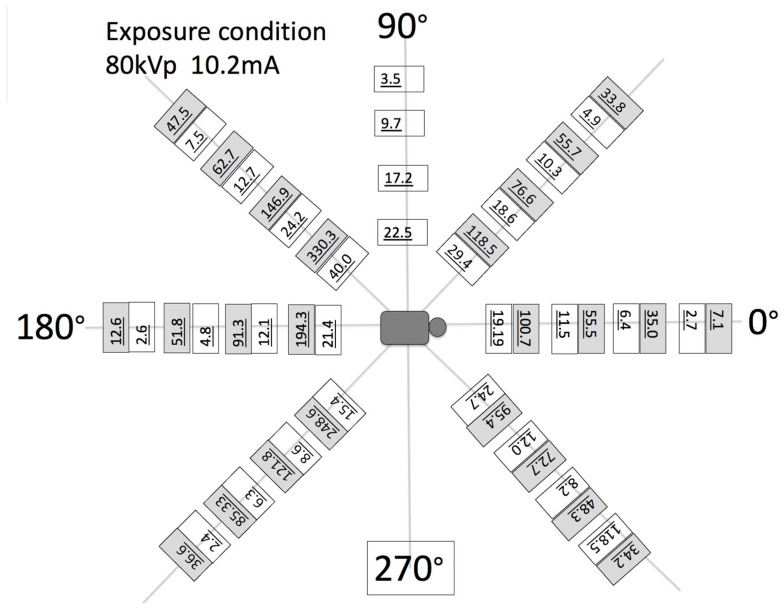
Scattered radiation dose from the robotic system in the 2D fluoroscopy mode. The scatter radiation was assessed in the surrounding area of the patient at equally spaced polar coordinate grids. For comparison the O-arm system (gray boxes) produced more scatter at almost all locations. The difference ranged from 1.9 times at the 0° position with a 100 cm distance to 20 times at the 270° position.

In Figure 16b the calibration frame allowing us to test for an unlimited choice of distances from the source to the phantom to the detector including the cross point of the mid-transverse, mid-coronal, and mid-sagittal planes. The source–detector unit in the frame could be configured in either a 2D fluoroscopic mode or CT mode. Since the source-to-isocenter distance of the robotic scanner varied, the dose was recorded for predefined fixed positions using the frame for consistency. We always ensured that the phantom was correctly aligned in all three planes (sagittal, axial, and coronal). For the pediatric head scans, for example, we positioned the 16 cm phantom on a table top. The predefined option “Chest” (80 kVp, 10.2 mA) was used because this option is the most frequently selected one for the pediatric scans. Several different source-to-detector configurations were investigated, as shown in Figure 17.

The measured doses of the robotic system at the specific locations (the isocenter in this experiment was always 65 cm from the source) on the pediatric phantom with protocol 80 kVp, 10.2 mA were as follows (values in μGy min^−1^): eye, 62; eye at 90°, 15.4; thyroid, 36.2; chest, 59.2; and gonad 16.4. Exposure was in the anteroposterior (AP) direction in all cases. For comparison, the largest effective dose burden for pediatric fluoroscopy as recorded elsewhere [5] for posteroanterior and lateral abdominal procedures was 0.2–1.1 mSv min^−1^, whereas procedures of the head resulted in doses of 0.02–0.08 mSv min^−1^ for fluoroscopy.

Figure 17 shows the measured dose of scattered radiation from the robotic system in the 2D fluoroscopy mode at different circular locations. When these data were compared to the O-arm system, it was observed that the O-arm produced more scatter radiation exposure at almost all locations compared with the robotic system. The difference ranged from 1.9 times at 0° position with a 100 cm distance to 20 times at 270° position with a 60 cm distance.

Another set of tests investigated the effect of the high-speed pulsed grid (BPPF) mode on the image quality, dosage results, and power output of the X-ray tube and generator. Some of the results of experiments case i to case vi, and Dose A to Dose D, i.e., the results of the pure pulsed and cine fluoro modes along with the system’s alternative new burst progressive pulse fluoro (BPPF) are shown in Figure 9. With the baseline standard pulse fluoro (case iv, no grid) for a generator setting of 100 kVp/25 mA/30 fps at 2.5 kW, the dosage reported was 1.9 R min^−1^. The six different tests cases were all recorded at 15 fps with frames lasting 5 ms each, and the time between frame being ~61 ms. It was clear that the use of a grid dramatically lowered the dose while image quality remained constant or even slightly improved. The first test (Figure 9b top) showed dosage of 1.9 R/min; this is the standard pulse fluoro (no grid). The second test (Figure 9b bottom) followed the same protocol, but a grid switch was used to remove the rise and fall of the waveform causing the dose to drop to 1.32 R/min. The third test (Figure 9a) described the effect of the BPPF mode of the robotic system, i.e., the high-speed pulsed grid (in BPPF, the rise and fall times of each pulse were removed and the X-ray was also turned on and off multiple times within each pulse). This mode synchronized the grid pulses with the robots’ camera/detector frame rate. The dose dropped to 0.72 R/min (a 62% reduction in dose from the non-gridded example). Using the same method, in 30/60/120/400 fps typical pulse fluoro, the system yielded even greater percentage savings in the order of 74–82%. We noticed that all three of the non-BPPF techniques had very similar image quality.

We also noticed that the speed-modulated grid image (BPPF) demonstrated another advantage. If we kept the dose at 1.9 R, the image quality was remarkably better, as shown also with the pediatric head phantom image in Figure 16a. These images in this configuration can then be described as cine quality with much higher diagnostic value at low dose, i.e., at standard fluoro dose rates, a unique finding for this system.

In addition to the reduction in dosage, the experiments (case (i) to case (vi)) also revealed another interesting aspect of the robotic system: cases (i) and (ii) (100 kVp/40 mA/30 fps configuration) required 4 kW to generate X-rays and 3 and 0 kW, respectively, in tube heat. Thus, they generated a total output power of 7 kW and 4 kW, respectively. In the next configuration of 100 kVp/25 mA/30 fps, cases (iv) and (v) required 2.5 kW to generate X-rays and 3 and 0 kW, respectively, in tube heat. Thus, they generated a total output power of 5.5 kW and 2.5 kW, respectively. The BPPF in the first configuration required 2 kW to generate X-ray and 0 kW in tube heat, whereas the respective numbers for the second configuration were 1.25 kW and 0 kW. Therefore, the total power output of BPPF was 2 kW and 1.25 kW, respectively, for the two configurations, suggesting significant (50–70%) reduction in heat generation by the X-ray tube.

Although the above exact test methodology was not followed for the detector option (c), we set up the detector at its high-speed data acquisition mode (600 fps) with the 40 mA/60 kVp configuration for comparison. The lowest detectable dose rate achieved was 13 nGyair s^−1^, which is over 400 times lower (from a dataset containing 30 frames) than typical medical diagnostic baseline, without sacrificing image quality. The difference is that the measurements were taken at a distance of 75 cm, and this was the lowest reading we observed so far in this system without deterioration in image quality. Additional dosage and diagnostic value experiments over- (Figure 10A,B) and under-exposed (Figure 10C) the scans of the pediatric phantom and were evaluated by a certified radiologist to systematize the correspondence between the tests’ gray-level histogram (brightness–contrast) and the diagnostic quality of the image. This analysis indicated that the BPPF X-ray generation mode (Figure 10D–F) always offered the optimal dose-to-image quality results.

The in vivo data in Figure 12 came from a 5-year-old patient that was scanned without anesthesia during the “golden hour” of what was later diagnosed as an arterial ischemic stroke incident (multifocal, with the patient exhibiting severe ataxia and acute hemiparesis). The quality of the image was critical as it revealed major multiple thrombosis at the basilar artery (arrow in Figure 12: the imaging revealed areas of chronic infarction in the left cerebellar areas and dissection of the distal basilar artery). Immediately after the diagnosis, a specific anticoagulant (coagulopathy workup) and thrombolysis treatment was prescribed. The dose for that robotic scan reached 6.2 mSv (Table 1), which is well within the mean size-specific dose estimate (SSDE) for the smallest patients in the literature (11.9 mGy) [19]. An MRI/MRA scan (Stony Brook Medicine SPIN—Circle of Willis (COW) spin—on a Siemens Magnetom Aera 1.5 T MRI scanner) (Figure 13) was also performed a year post-stroke. That MRA scan was a specialist magnetic resonance angiography scan which examined specific blood vessels at the base of the brain, a connection of several main arteries which supply the brain with blood containing nutrients and oxygen. The MRA scan showed an increased level of angiogenesis and full recovery in and around the patient’s region of brain stroke.

**Figure 18:**
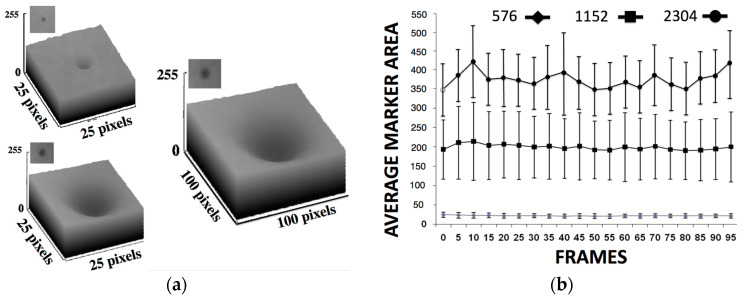
(**a**) Tantalum marker’s X-ray signature surface plot for 576 × 576, (**top**), 1152 × 1152 (**bottom**), and 2304 × 2304 pixels (**right**). Note how resolution affects the size of the marker’s X-ray signature. At the highest resolution, almost 5 times the number of pixels are available to track using the market tracking software. (**b**) Higher average marker area associated with the higher resolution images when summing 100 frames of acquisition.

The in vivo soft and hard tissue kinematics (Figure 3) tracking used external and internal fiducial markers (0.1 mm tantalum beads) from the patient’s segment or phantom as described in stereovideoradiography approaches involving the same device that were published elsewhere [38,39,43,45,49]. The six-DOF motion parameters of a fast moving target (marker at bone of athlete running at 3 m/s and jumping lower-extremity amputee patient) were determined with a high accuracy of 10 μm for translation and 0.02° for rotation (Table 1). This accuracy was observed at the highest resolution option (2304 *×* 2304 pixels) of the imager’s high-speed detector camera (Figure 18). This resolution set the capacity for motion during each video frame exposure to 1 mm or less for object velocities up to 10 m/s based on the average marker area of the marker’s X-ray signature.

Figure 3a shows a high-speed stereovideoradiography cardiac angiogram (the left image is a high-speed fluoro frame from the robotic scanner CATHLAB mode) and dynamic assessment of arthrokinematics; the right image shows a high-speed frame of a dynamic knee study with the patient running at speeds exceeding 3 m/s. Note the bone-embedded tantalum beads that were used to validate the marker and markerless tissue kinematics tracking methods we presented elsewhere [39,45]. The trail of the position of the marker shows how its displacement was tracked in one of the two stereo views; Figure 3b shows examples of scanning adopted from past studies [39,45,48] presenting a jumping below-knee amputee patient (top left) surrounded by the four robot scanner (two emitters and two detectors) and the two corresponding videoradiography views recorded during the jumping task (b, far right); the bottom left image presents the tracking of the kinematics of a patient’s knee internal prosthesis. The tracking of the 3D displacement is always indicated with multiple points that form a colored line indicating the time course of the displacement. In that sense these datasets can be used to calculate the velocity and acceleration at all these instantaneous positions. It should be stressed that the system’s open gantry motion-correction configuration does not limit the overall FoV. This makes scanning large non-axisymmetric parts of the human anatomy during increased ranges of movement and load bearing without anesthesia possible.

## 4. Discussion

The current study presented different types of comparative data so that the performance of a new robotic imager could be characterized for the first time with respect to similar technologies. We tuned all the tests towards demonstrating the robotic multimodal imaging system’s unique features and how it could revolutionize pediatric imaging, especially in terms of lowering radiation exposure and eliminating anesthesia. For this goal, a series of new calibration experiments were designed to characterize dosage options in relation to improved image quality and the identification of soft- and hard-tissue boundaries during static imaging and imaging during patient movement. However, as some methods presented here were devised for the first time, we did not produce datasets with enough depth and in high quantity to allow statistical analyses. The work did not aim to show significance but to indicate the main trends of the various novel calibration methods with known phantoms and calibration tools for a very different imaging system architecture that could not use previous conventional calibration methodologies. We believe, however, that the information presented here is valid for all future investigations of similar robotic hybrid imagers. Usually, the performance of scanners includes separate estimations for single-source, cardiac, dual-source (obese), dual-energy modes for the devices having fixed geometry (i.e., donut-shaped or gantry/ring-fixed), which was not the case in the robotic scanner. The purpose of this early acceptance testing was twofold: (1) to compare some of the performance metrics with expected values, provided either from the manufacturer or from published values; and (2) to establish baseline values for comparison with future measurements, particularly for pediatric imaging.

It is obvious, however, that obtaining a complete set of performance metrics can be difficult, especially with this new generation of imaging devices that have a very different system architecture which makes these comparisons very challenging. The large number of possible tube/detector configurations, specialty collimation technology, and the resulting geometrical parameters for optimized volume reconstruction available on this robotic scanner presents a unique challenge to the medical physicist for acceptance testing and routine quality assurance measurements. This is compounded by the fact that some combinations are not limited to only spiral or sequential acquisitions. This is especially problematic for Computed Tomography Dose Index (CTDI) measurements, where variable spiral acquisitions or completely random emitter-to-detector trajectories may not have comparable sequential acquisitions. This is because the system uses different robotic arm trajectories to meet the requirements of the size of the patient to be scanned. Additionally, the second or third X-ray tube, typically referred to as the “B- or C-tubes”, can be operated independently of the primary X-ray tube (the “A-tube”). This implies that the physicist must make reasonable assumptions in order to acquire clinically meaningful CTDI measurements on the system.

This robot multimodal complexity led us to choose a very simplified experimentation method. When the detector configuration used for clinical applications cannot be matched for a CTDI measurement, the configuration with the next closest total detector collimation should be used, which is consistent with the requirements for American College of Radiology (ACR) accreditation dose measurements. This means step-by-step experiments where only one tube was tested before the second tube and detector were added on and so on, a practice that will be repeated in the future evaluations of this scanner. For CTDI measurements, the CTDI from the B-tube must be deduced by measuring the CTDI from the A-tube and the A+B-tubes (both tubes used simultaneously at the same kV). This was the case for all the experiments here, and attention was given when the combined results were reported. The fluoroscopy mode allowed one-tube-only scans and was tested separately. Therefore, this work lists scan parameters for various tests used for acceptance testing and routine quality assurance testing. The choice of tube-current values was within the clinically useful range and will remain consistent for all future measurements. Detailed instructions for common tests were not provided here, as this information is widely available [7]. Additionally, as the positional accuracy of the robotic arms was very high (microlevel), tests that were independent of the CT scan parameters, such as table incrementation, were not investigated.

Dynamic range (related contrast), noise (CNR), resolution, distortion, scatter, focal spot, artifacts, exposure, and exposure time accuracy and constancy were all considered when reporting the results. The work, however, did not report on limiting resolution center, sharpness corner, optoelectronic conversion function, binning effect, sensor uniformity, and shatter lag. The calibration processes utilizing three types of different detector technologies suggested that the option (c) detector (perovskite) had the best performance for scatter artifacts, contrast, and spatial resolution. These results were consistently improved with scatter correction (SC) and beam-hardening correction (BHC) for all detectors, while they were also performed at much lower dose parameters (up to 50% less, which is expected to impact overall dose characteristics for pediatric imaging). The bowtie filter effect was effective in reducing BH artifacts when the SC increased noise. The results with the detector (c) presented the highest resolution (~9 lp mm^−1^, double compared to the other two detectors) under a remarkably low X-ray dose of ∼56 μGyai. This dose was half of the X-ray dose observed in the other two detectors for high-diagnostic-quality images. This finding alone is rather impactful when pediatric imaging is concerned. The system’s highest dynamic translational and rotational accuracy values were 10 µm and 0.02°, respectively. In addition, a considerable difference (up to 50%) in dose–area product DAP and air kerma was observed between detectors (a), (b), and detector (c).

In all the experiments performed here, radiation dose never came close to 50 mSv, which is the dosage in the majority of pediatric CT scans in the state of the art and has been reported as statistically significant for an increased risk of fatal cancer. In fluoroscopy mode, head scanning resulted in doses of 62 μGy min^−1^, well within the ranges reported in the literature (0.02–0.08 mSv min^−1^) for interventional radiology procedures [5]. This dose profile was a little higher for the musculoskeletal kinematics stereovideography assessment of the torso and lower extremities (athlete, amputee) for a longer duration of exercise.

One of the major takeaways from this study is a systematic series of new dosage reduction methodologies that can be critical for some sensitive populations. These methods include special collimation options, dynamically adjusted focal spots, and variable exposure and dynamic alteration of the trajectories of emitters and detectors for optimal imaging. The measured doses of the robotic system at specific locations in the head and body (with the isocenter always being 65 cm from the source) on the pediatric phantom were always well within or better compared to the age-based results reported elsewhere [5]. This finding is again expected to improve the overall system’s impact on pediatric patients.

Similarly, the measured doses of scattered radiation from the robotic system in the 2-D fluoroscopy mode at different circular locations were between 2 to 20 times lower than comparable values of similar systems (Figure 17). Careful interpretation of these results must be exercised though, as the difference could be attributable to a number of parameters. The comparable O-arm “Thorax” option produces more than double the dose from the robotic “Chest” option that it was compared to. Data from the O-Arm were chosen for comparison due to its large gantry that resembled some of the scanning options offered by the robotic scanner. Furthermore, the beam area of the X-ray source was wider in the O-arm, as it did not have the specialized collimation offered in the robotic system (Figure 4); thus, it could cause more scatter from the phantoms and neighboring objects. The radiation doses decreased according to the increasing distance from the central radiation source. The tests on the robotic scanner did not include PA positioning, as the AP position has been reported [4] to yield higher entrance surface doses to the eyes, thyroid, and breast than the standard PA configuration. These PA experiments need to be performed in a future study to compare with these findings. It can be concluded that this tool could have a positive impact on the way interventional pediatric radiology is performed in the future.

When a simplified direct comparison of dose and image quality was performed, investigating pure pulsed, cine fluoro, and the system’s alternative new burst progressive pulse fluoro mode (BPPF), it was clear that the use of a grid dramatically lowered the dose (by 62%) while image quality remained constant or even slightly improved. Using the same method, in 30/60/120/400 fps typical pulse fluoro, the system yielded even greater percentage savings in the order of 74–82%. A potential very important result for pediatric fluoroscopy here is that if we keep the dose at 1.9 R, the image quality is remarkably better. This means that the device offers cine quality fluoroscopy options with high diagnostic value at low dose rates and for potentially larger durations making it more applicable for interventional radiographic and intraoperative navigation procedures.

The tomosynthesis scans using a dynamically adjusted focal spot (ranging from 0.1 to 0.02 mm) allowed 3D reconstructions with 40 μm using long, wide, and appropriately elliptical trajectories of the emitters (the detectors had to be stationary) only possible with this robotic system. Although narrow with respect to the FoV, these microlevel scans are free of the noise that CT scans have at higher binning options [53]. However, more work is required to study this effect of tomosynthesis. Another limitation in this study is that it did not take into account the binning level and the level of optical magnification at the detector for each modality, and their results on the resolution and FoV were therefore not reported.

The next set of experiments (case (i) to case (vi)) also revealed another interesting aspect of this technology. The total power output of the BPPF mode resulted in a 50–70% reduction in heat generation by the X-ray tube. This means less stress on the generator which prolongs useful life and allows longer use during procedures. This aspect presents a sustainability profile that the one-device–multiple-modalities principle can also be claimed to possess inherently. The robotic scanner saves the hospital a significant cost in capital expenses and operating expenses (energy savings) as it saves time (no special preparation is required between modalities) and space, requiring no additional rooms that all these modalities would have to be housed in. An appropriate cost–benefit analysis is required, however, to evaluate these factors before final conclusions.

The lowest detectable dose rate using the phantom 3D experiments (6.1 mSv) was achieved with detector option (c), which was characteristically lower than the typical medical diagnostic baseline (above 11 mSv). These experiments also helped start a systemization for the robotic scanner by means of “standardizing” its brightness–contrast performance and how this relates to diagnostic quality. This in turn could inform a potential AI-based, radiologist-evaluated optimal dose-to-image algorithm in the future.

Finally, these open gantry motion-correction automated systems do not limit the overall FoV. As a result, they make scanning large non-axisymmetric parts of the human pediatric (or adult) anatomy during an increased range of movement without anesthesia possible. Therefore, in an in vivo 5-year-old patient, a no-anesthesia 3D scan (6.2 mSv) was possible during the “golden hour” and helped diagnose an intracranial ischemic stroke incident with a major basilar artery thrombosis. Immediately after the diagnosis, a specific anticoagulant and thrombolysis treatment was prescribed leading to expedited recovery of the patient. Data from an MRI/MRA scan that was also performed a year post-stroke indicated full recovery and increased angiogenesis in the region of the stroke. This anesthesia-free scanning is extremely important as the effect of anesthesia for this kind of patient varies from neurological acute side-effects post-anesthesia administration to neuronal degeneration, long-lasting cognitive and behavioral deficiencies, paraplegia, and even 16% or more mortality. It is well known that the Acute Ischemic Stroke (AIS) pediatric patient population has a higher incidence in newborns than in older children (1/3500 live birth versus 1–2/100,000 per year) and that randomized clinical trials assessing safety and efficacy of anesthesia during imaging, thrombolysis and/or endovascular treatment were never performed in these children [20,28,29]. Past studies [8,9,12,13,14,15,16,17,18,19,20,21,22,23,24,25,26,27,28,29,30,31,32,33] have reported the widespread use of X-rays, and its statistically significant influence on the increased risk of fatal cancer from low-dose radiation in the range of 50 to 100 mSv (mGy) which is the dosage in the majority of pediatric CT scans in the state of the art. It was noted that a single CT of the abdomen could provide a dose of 11 mSv. If there were three phases in this examination, the actual dose would be 33 mSv. If this child was one of the 30% who had three or more examinations, the lifetime dose would at least 100 mSv, clearly in the range of doses associated with the induction of fatal cancer [21,25].

It was also reported that the congenital heart disease (CHD) patient population (its prevalence being 8 per 1000 live births [30]) continues to be at significant risk for neurodevelopmental sequelae throughout their life course, some of which due to anesthesia and high-dosage imaging [37,40,41]. The literature shows that the variation in patient dose due to inappropriate technique or failure to adapt imaging protocols from adults to children to account for both pediatric diseases and different patient sizes is not appropriate and requires the optimization of protection. The results presented here can inform the clinical integration of such a hybrid imager in pediatric diagnostics. However, we should note the limited in vivo sample size (case study) and the future requirement for additional validation in patient groups with more diversity.

It is clear that much more work is required in the future to characterize these hybrid systems. Their capacity to automatically and dynamically alter the focal spot and magnification with respect to amplitude, exposure, and the position of the patient with respect to the emitter and the detector opens a new paradigm in designing imaging protocols. It also complicates the task of the medical physicist. This study’s limitations include the lack of attention to the effects of quantum mottle, viewing distance, line of orientation, the frequency dependence of attenuation, and multi-scattering effect, among others. These parameters, when clearly studied, are expected to further enhance the pediatric imaging protocols as they are related to dose reduction and image quality. We did not report on dynamic range, noise resolution, and potential sources of distortion. These effects should also be studied more, along with the limiting resolution center, sharpness corner, shutter lag, and focal spot quantification. Future tests ought to include white balancing and a detailed optoelectronic conversion function, sensor uniformity, and exposure and exposure time accuracy and constancy. Although it was not within the scope of the present study, it should be noted that the optimization of these parameters can be performed with the help of an AI suite that can continuously evaluate each system and organize the data.

Patient-specific controlled radiation dosage with finely tuned calibrated devices such as the one presented here, which can scan a patient without anesthesia, has always been an important issue in radiological scans for patients, surgeons, and the operating theatre staff. With every new system in the market, the main technical challenges concentrate on the capacity to standardize and ease the calibration procedures. Although the system appears to have high complexity, it proved to be very ergonomically and logistically friendly to the user and the patient, mainly due to its open architecture, scan duration results (Table 1), and level of automation. Its scalability for widespread adoption ought to be studied in a separate review once more data from early adopters are available. The study suggests that the optimization of patient radiation dose due to appropriate calibration techniques or the adaptation of imaging protocols from adults to children is possible. This optimization ought to account for both pediatric diseases and different patient sizes, but it could also be enhanced with a modular automation-driven system and the input of AI. These image quality and dosage challenges burden the brain trauma, cancer, and cardiovascular pediatric patients as they often require multiple revisions and surgeries with the disadvantage of the accumulative dose effects of additional radiographic imaging. Each pediatric tertiary center should therefore activate adequate institutional and detailed device calibration protocols for the optimization of diagnostic work-up and treatments. Automated systems might prove to be better enablers for this type of calibration work.

This new generation of imaging devices might prove to be the unique tools to utilize in randomized clinical trials assessing safety and efficacy of anesthesia during imaging, brain trauma, stroke (the sixth leading cause of death in children), thrombolysis, and/or endovascular treatment that to this date have never been performed in pediatric patients.

## 5. Conclusions

Performance testing of robotic hybrid scanners presents additional challenges as there are many different modalities, scan configurations, and options available, and the physicist must determine which parameters are essential for characterizing and monitoring the clinical performance of the scanner. Our experience has shown that there may be clinical configurations that cannot be readily tested using conventional calibration techniques. In addition, as this technology continues to mature, new features and options are likely to emerge. Information gleaned from our new calibration and clinical protocols can be highly beneficial, particularly for the sensitive pediatric patient population where dosage and anesthesia introduce high risks. The robotic open gantry motion-correction automated systems do not limit the overall FoV; this makes scanning large non-axisymmetric parts of the human pediatric (or adult) anatomy during increased range of movement without anesthesia possible. The optimization of patient radiation dose with respect to image quality due to appropriate techniques or the adaptation of imaging protocols from adults to children to account for both pediatric diseases and different patient sizes showed encouraging results with this new hybrid robotic scanner. The potential clinical impact, especially regarding anesthesia-free low-dose pediatric imaging is the most important finding of this work. The effects of the power, the geometry–angle of penetration of the X-ray signal, and its exact diffractive/absorbing/scattering effect from individual hard and soft tissues should be constantly reassessed for these new generation of scanners. Future research directions, including possible refinements in these hybrid robotic scanners’ calibration and validation studies, are required to complete their characterization.

## 6. Patents

A patent resulted from the work presented here and is reported in reference [46].

## Figures and Tables

**Table 1:** The relationship between the high-performance modalities of the robotic scanner and several parameters such as resolution, min and max scan duration and frame rate during the acquisition, dose ranges for chest, gonad, and head scans, and source-to-object (SOD), source-to-imager (SID), reconstructive conical volume diameter (VD), and conical height volume (VH) as well as focal spot.

Parameters Modality	Resolution /Type of Reconstruction	Min–Max Scan Duration/Min- Max Frame acq. Rate	Dose Chest/Gonad/Head Imaging (μGy min^−1^)	SID/SOD/VD/VH/FS (mm)
2D radiography (see Figure 5, Figure 6, Figure 11 and Figure 16)	See detector a, b, c resolution	1 s to 2 min/1–360	~50/50/50	SID 50–1100/ everything else is variable
3D CT (Figure 2 and Figure 12)	80–200 μm full CT, 3x mode CT, half-beam mode, stacked-volume mode	6 s to 4 min/40–5000	~50–500/16.4–200/62–120 Total dose in full in vivo high-res. CT: 6.2 mSv	700/480/240/0.06
3D tomosynthesis (see Figure 2 and Figure 11). Optical mag., x4	10–40 μm fixed detector, moving emitter	6 s to 2 min/5–5000	~30/16.4/34	50–1100/variable SOD/variable VD and VH/0.02–0.2
3D dynamic imaging stereovideography (Figure 5, Figure 6 and Figure 9)	20–200 μm Static or moving image intensifiers with high-speed cameras	1 s, as required by IR/10–10000	0.02–1.1 mSv min^−1^/0.08–0.9 mSv min^−1^/0.02–0.08 mSv min^−1^	50–1100 /10–500/variable VD and VH/0.02–0.2
3D “microCT” optical and geometrical magnification x4 (Figure 2 and Figure 13)	0.02 mm Emitter/detector rotation only	1 min to 5 min or as required/100–5000	0.2–5 mSv min^−1^/not available/0.2–5 mSv min^−1^	50–100 /10–50/variable VD and VH/0.02

## Data Availability

Restrictions apply to the datasets. The datasets presented in this article are not readily available because of privacy issues, e.g., the data are part of an ongoing study. Also, technical/time limitations contribute to these restrictions.

## Abbreviations

The following abbreviations are used in this manuscript:

CT	Computed tomography
MRI/A	Magnetic Resonance imaging/Angiography
FoV	Field of view
HMI	Human–machine interface
EoAT	End-of-arm toolkits
SOD	Source-to-object distance
SID	Source-to-imager distance
OID	Object-to-imager distance
PLC	Programmable Logic Controllers
SNR	Signal-to-noise ratio
CNR	Contrast-to-noise ration
IGZO	Indium gallium zinc oxide
CMOS	Complementary metal–oxide–semiconductor
DC-TDI	Direct conversion, Time Delay Integration
PAN	Panoramic
CATHLAB	Catheterization laboratory
AIS	Acute Ischemic Stroke
CHD	Congenital heart disease
ESPR	European Society of Pediatric Radiology
CAD	Computer aided design
AI	Artificial intelligence
2D	Two-dimensional
3D	Three-dimensional
4D	Four-dimensional
VH	Volume height (of cone)
VD	Volume diameter (of cone)
FS	Focal spot
LDDR	Lowest detectable dose rate
BPPF	Burst progressive pulse fluoro mode
EDR	Extreme dynamic range
PA	Posterior-anterior
SC	Scatter correction
BHC	Beam-hardening correction
ACR	American College of Radiology
CTDI	Computed Tomography Dose Index
DAP	Dose–area product
ALARA	As low as reasonably achievable
DOF	Degree of freedom
